# Central Adiposity Assessed with Body Roundness Index and Mortality: The Seguimiento Universidad de Navarra Prospective Cohort

**DOI:** 10.3390/geriatrics10060135

**Published:** 2025-10-23

**Authors:** Ligia J. Dominguez, Carmen Sayon-Orea, Estefania Toledo, Maira Bes-Rastrollo, Carolina Donat-Vargas, Mario Barbagallo, Miguel A. Martínez-González

**Affiliations:** 1 Department of Medicine, University Kore of Enna, 94100 Enna, Italy; 2 Geriatric Unit, Department of Internal Medicine and Geriatrics, University of Palermo, 90127 Palermo, Italy; mario.barbagallo@unipa.it; 3 Department of Preventive Medicine and Public Health-IdiSNA, University of Navarra, 31008 Pamplona, Spain; msayon@unav.es (C.S.-O.); etoledo@unav.es (E.T.); mbes@unav.es (M.B.-R.); 4 CIBER Fisiopatologia de la Obesidad y Nutrición (CIBERobn), Instituto de Salud Carlos III, 28029 Madrid, Spain; 5 Public Health Institute, 31003 Navarra, Spain; 6 Department of Nutrition, Food Sciences and Gastronomy, Faculty of Pharmacy and Food Sciences, INSA-University of Barcelona, 08028 Barcelona, Spain; carolina.donat@ub.edu; 7 Epidemiology and Public Health Networking Biomedical Research Centre (CIBERESP), Instituto de Salud Carlos III, 28029 Madrid, Spain; 8 Unit of Cardiovascular and Nutritional Epidemiology, Institute of Environmental Medicine, Karolinska Institutet, 17177 Stockholm, Sweden; 9 Department of Nutrition, Harvard TH Chan School of Public Health, Boston, MA 02115, USA

**Keywords:** obesity, adiposity, body mass index, mortality, longitudinal

## Abstract

**Background/Objectives:** Obesity is currently a global pandemic and a major risk factor for the development of chronic disease and increased mortality. Common methods used to define obesity, such as body mass index (BMI), do not accurately reflect body fat content or distribution. **Methods:** We investigated the prognostic significance of the body roundness index (BRI) on incident death in 12,642 participants (60.2% women, mean age: 39, standard deviation (SD): 12 years) from the “Seguimiento Universidad de Navarra” prospective cohort and compared it to waist-to-height ratio (WtHR) and waist circumference (WC). Participants were monitored through biennial questionnaires. The mean of the baseline BRI was 3.6 (SD: 1.4) units. Multivariable-adjusted Cox models were used to estimate hazard ratios (HR) and confidence intervals (CI) of death. **Results:** Over a median follow-up period of 11.5 years, 380 participants died (absolute mortality rate 1.74 × 10^−3^). In multivariable-adjusted models, higher quartiles of BRI were significantly associated with all-cause death, specifically in those ≥ 60 years (Quartile 4 vs. Quartile 1: HR 1.64; 95% CI: 1.00, 2.70). Considering the whole group (all ages), each 2-unit increase in BRI was linked to a 21% higher all-cause mortality risk in both men and women. This association was even stronger for participants aged over 60 years (multivariate adjusted HR for 2-unit BRI increase: 1.31; CI: 1.00, 1.72), while it was not significant when considering only those under 60 years. The associations of z-WtHR and z-WC with incident mortality for all participants were also significant in the fully adjusted model (HRs: 1.14; CI: 1.01, 1.27, and HRs: 1.16; CI: 1.04, 1.30, respectively). Mortality associations assessed using the BRI, WtHR, and WC were superior to associations based on the BMI. **Conclusions:** BRI shows a linear link with all-cause mortality in healthy adults ≥ 60, while WtHR and WC were also mortality predictors. Thus, lower central fat may help reduce early death risk.

## 1. Introduction

Obesity is a growing global epidemic and key public health issue. Around two-thirds of adults in Western countries are currently classified as overweight (body mass index [BMI] > 25) or obese (BMI > 30). Since 1975, obesity rates have tripled, driven by poor diets and sedentary lifestyles [1]. Excess fat accumulation—general or ectopic—increases the risk of adverse health outcomes [2].

Excess body fat is strongly linked to reduced life expectancy and a higher risk of numerous chronic non-communicable diseases, including type 2 diabetes, hypertension, stroke, coronary heart disease, fatty liver, chronic kidney disease, dementia, sleep apnea, and various cancers [3]. Although BMI has continued to rise globally and obesity’s role in chronic disease risk is well established, its association with long-term all-cause mortality remains uncertain [4,5,6].

Indeed, estimates of mortality risk linked to overweight and obesity vary widely across studies. While some research suggests that only severe obesity raises mortality, that mild obesity (BMI 30.0–35.0) has little impact, and that overweight (BMI 25.0–30.0) has even been linked to lower mortality [7,8], these findings have faced criticism [9]. Other studies, adjusting for bias, show a near-linear relationship between elevated BMI and mortality, with overweight and obesity potentially accounting for over 15% of adult deaths in the U.S. [10,11].

Research has increasingly linked visceral fat to higher mortality risk [12,13]. BMI alone may not reflect health risk, as fat percentage and distribution vary at the same BMI [14,15]. One study found that mortality risk in BMI ≥ 30 kg/m^2^ ranged from 21–108% vs. normal weight, but BMI wasn’t an independent predictor, underscoring the importance of body composition and comorbidities [5]. In a large cohort study over 5.7 years, Lee et al. [16] reported that the visceral-to-subcutaneous fat ratio was more strongly associated with all-cause mortality than BMI. Moreover, BMI’s predictive value outside the normal range varied with other anthropometric and clinical factors [17,18].

Methods like air displacement plethysmography, isotope dilution, DXA, skinfolds, bioelectrical impedance, MRI, and CT can assess body fat and its distribution but are costly, complex, and not practical for routine clinical use [19]. As a result, alternative indices with stronger correlations to actual fat levels have been developed [20].

Waist and hip measurements, and their ratios, are useful indicators of body shape and fat-related risk [21], but they omit height, possibly overestimating risk in taller individuals. To address this, Thomas et al. developed the Body Roundness Index (BRI) in 2013 [22], which uses elliptical body models and eccentricity to estimate visceral and total body fat. BRI incorporates waist circumference, weight, and height for a more accurate assessment of fat distribution [22]. Ashwell et al. had previously proposed the waist-to-height ratio (WHtR) as a marker of abdominal obesity [23]. Compared to previous measures, WHtR showed similar or slightly stronger associations with cardiovascular disease (CVD) and type 2 diabetes risk [24]. This may be partly due to the link between shorter stature and increased CVD risk [25].

Since its introduction, BRI has shown strong clinical potential. A systematic review found it superior to traditional measures in predicting metabolic syndrome [26]. A study of over 17,000 adults in Eastern China identified BRI as a better marker of cardiometabolic risk [27]. NHANES data (15,000+ participants) also showed that BRI had a stronger link to frailty than BMI, both showing a U-shaped relationship with all-cause mortality in frail individuals [28]. However, research on BRI and mortality is still limited, particularly in Mediterranean populations with distinct dietary patterns.

Based on this background, we aimed to prospectively examine the relationship between body fat distribution, as assessed by the BRI, WtHR and waist circumference (WC), and the risk of death, in comparison to BMI, within the Mediterranean population of the SUN (“Seguimiento Universidad de Navarra”) longitudinal study.

## 2. Methods

### 2.1. Study Design and Participants

The SUN project is an ongoing, multipurpose, permanently open dynamic cohort study. Since December 1999, graduates from the University of Navarra and other Spanish universities have been invited to participate. It collects updated data every two years on diet, lifestyle, and other health risk factors, as well as on medical conditions present at the start of the study and those identified during follow-up, aiming at assessing how diet and lifestyle influence the prevention of non-communicable diseases [29]. More detailed information about the study design and methods has been previously published [30,31,32]. The baseline questionnaire collects data on sociodemographics, anthropometrics, diet, lifestyle, clinical history, medications, and personality traits. Self-reported data (e.g., anthropometrics [33], physical activity [34], hypertension [35], metabolic syndrome [36], and depression [37]) have been validated. Waist and hip circumferences (at 6 and 8 years) are self-measured using mailed tape and instructions.

The data used in the analyses of the present study belong to the SUN project database updated until 30 April 2024, which included 23,321 participants. We excluded 727 participants whose follow-up period was shorter than six years and nine months, accounting for the lag time in returning the questionnaire established in the protocol of the cohort (to allow for returning the first 2-year follow-up questionnaire); we also excluded 450 participants with total energy intake outside the predefined limits (p1 p99), 5183 without follow-up and 4207 without questionnaires at six and eight years (in which waist circumference was measured), and 112 with missing data for waist circumference. Supplementary Table S1 shows the characteristics of included and excluded participants. As seen in the table, they were very similar in almost all variables, confirming that exclusion did not influence the findings.

**Figure 1:**
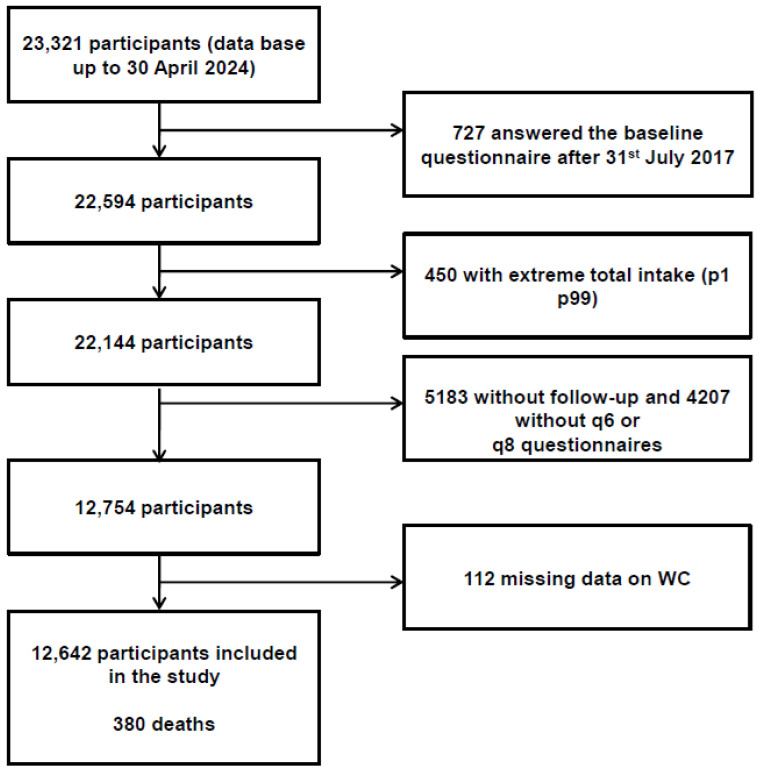
Flowchart depicting the selection process among participants of the SUN project to be included in the present analyses. WC: waist circumference.

The final sample for longitudinal analysis consisted of 12,642 participants, with an overall retention rate of 77% (Figure 1).

### 2.2. BRI Definition

We calculated the BRI with the equation proposed by Thomas et al. [22]:

$$BRI=364.2-365.5\;\times \sqrt{1-\frac{{\left(\frac{WC}{2\pi }\right)}^{2}}{{\left(0.5\;height\right)}^{2}}}$$

A measuring tape was sent to all participants for the questionaries at six and eight years of follow-up, along with detailed instructions on how to measure their own waist. The validity of these self-reported measurements has been previously assessed [36]. Due to the lack of a reference range, BRI was categorized into quartiles to explore the association with all-cause mortality.

### 2.3. Outcome

We tracked each new death in the cohort through continuous and active follow-up with all participants. Every year, we reached out to each participant multiple times, requesting updates on any changes to their postal addresses, and provided three alternative addresses for each individual. Additionally, telephone numbers and email addresses were used as backup methods in case postal contact failed. The alumni associations of the University of Navarra, along with other professional organizations, also played a key role in locating participants who did not respond to follow-up questionnaires. Most (over 85%) of the deaths in the cohort were reported by next of kin, professional associations, or the postal service. To verify and complete the mortality data, including the cause of death, we checked the National Death Index every year to confirm the vital status of our participants.

### 2.4. Ethical Principles

All participants were provided with detailed written information regarding the data requested in the subsequent questionnaires, the future feedback they would receive from the SUN project research team, and the measures in place to protect their privacy regarding the information they provided. They were also informed of their right to decline participation in the SUN longitudinal project and the option to withdraw their consent at any time without facing any repercussions, in accordance with the ethical guidelines set forth in the Declaration of Helsinki for medical research involving humans. The completion and return of the first questionnaire at baseline, on a voluntary basis, was considered as informed consent, as approved by the Institutional Review Board of the University of Navarra (Project identification code 2001_30).

### 2.5. Other Covariates

For the multivariable statistical models, we also included additional covariates such as age, sex, marital status, smoking habits (categorized into 3 groups: never, former and current smokers), lifetime exposure to tobacco smoking (pack-years, continuous), years of university education, between-meal snacking, adoption of special diet, prevalent hypertriglyceridemia, prevalent hypercholesterolemia, prevalent diseases (cancer, diabetes, cardiovascular disease, all of them with very low prevalence at baseline), siesta, adherence to Mediterranean diet [38], television watching (h/d), total energy intake, leisure-time physical activity (METs-h/wk), and health-conscious score (defined as number of medical check-ups including 11 items). Physical activity was assessed using a validated questionnaire, with results validly compared to objective measurements from a triaxial accelerometer (RT3 Triaxial Research Tracker) (Spearman correlation coefficient of 0.51; *p* < 0.001) [34], and expressed in metabolic equivalent tasks (METs-h/week; calculated by multiplying the time spent in each activity by its usual energy expenditure) [39]. BMI was calculated based on self-reported weight and height, which were previously validated in a subsample of the SUN cohort. The mean relative error in self-reported weight was 1.45%, and the correlation coefficient between measured and self-reported weight was 0.99 (95% confidence interval (CI) 0.98–0.99) [33].

### 2.6. Statistical Analyses

The baseline characteristics of the sample, including means and SD for continuous variables and proportions for categorical variables, were calculated across the four quartiles of BRI. The follow-up time for each participant was determined by the period from the date they returned the sixth or eighth questionnaire to the date of death or the date of the last questionnaire, whichever occurred first. To examine the relationship between increasing BRI and the risk of incident death, we performed Cox regression analyses with age as the underlying time variable and estimating Hazard Ratios (HRs) and 95% confidence intervals (CIs). Death cases were calculated across the quartiles of the BRI, with the lowest quartile serving as the reference group. The proportional-hazards assumption was assessed using Schoenfeld residuals after fitting the model.

We employed a sequential modeling approach with progressively comprehensive adjustment for potential confounders. Thus, we initially estimated HRs without any adjustments (crude). Model 1 included HRs adjusted for age and sex, and Model 2 adjusted for factors in Model 1 plus marital status, years of university education, smoking status (never, former, or current smoker), pack-years of cumulative cigarette exposure, leisure-time physical activity (quartiles, METS-h/week), television watching (hours/day, continuous), total energy intake (quartiles, kcal/day, continuous), adherence to the Mediterranean diet (in categories: low 0–3, medium 4–6 and high 7–9 scored 0 to 9 points based on reference [38]), between-meal snacking, special diet adoption, prevalent hypertriglyceridemia, prevalent hypercholesterolemia, prevalent diseases at baseline (cancer, diabetes, cardiovascular disease), siesta, and health-conscious score.

The median BRI quartiles were treated as a continuous variable to assess the significance of a linear trend. We also calculated multivariable-adjusted HR estimates for the association between a 2-unit increase in the BRI and the incidence of death. These analyses were performed for the total population as well as for those older and younger than 60 years. We also calculated the risk in other age-groups (<40, 40–50, 50–60, and >60 years). Additionally, we performed analyses on the risk of incident death for each one SD (z-BF) increase in BRI as a continuous variable, separately for the total population and for participants older and younger than 60 years.

To assess the robustness of our findings, sensitivity analyses were conducted. We estimated fully adjusted HRs comparing the highest and lowest quartiles of BRI and their association with incident death, both for the whole population and for participants older than 60 years. These analyses systematically evaluated the stability of our results under various conditions: changing allowable energy limits compared to usual (<500 or >3500 kcal/day for women and <800 or >4000 kcal/day for men), including only never smokers, excluding participants with prevalent hypertension at baseline, excluding participants with prevalent diabetes at baseline, excluding participants with prevalent cancer at baseline, excluding participants with hypertriglyceridemia at baseline, and excluding participants with prevalent hypercholesterolemia at baseline.

Receiver-operating characteristic (ROC) analyses were conducted to evaluate the discriminative power of BRI and BMI in predicting death, with the roccomp Stata command used to compare the statistical significance of the area under the curve (AUC) values for BRI and BMI. Multicollinearity was tested using variance inflation factor (VIF). All analyses were performed using Stata software (version 16; Stata Corp. 4905 Lakeway Drive, College Station, TX 77845, USA). A two-tailed *p*-value of less than 0.05 was considered significant. Values presented in the text are means ± standard deviations (SDs) unless stated otherwise.

## 3. Results

### 3.1. Participant Characteristics

Over 218,319 person-years of follow-up (median follow-up: 11.5 years; IQR: 7.9–14.7 years) from 2006 to 2024, we identified 380 new deaths in the SUN cohort (absolute mortality rate 1.74 × 10^−3^). Table S1 presents the baseline characteristics of the participants, including demographic, anthropometric, and lifestyle factors, categorized by quartiles of BRI and by categories of BMI (Table 1). Among all participants, both men and women in the highest quartile of BRI were more likely to be older, married, smokers, and had higher BMI, a greater prevalence of chronic diseases (diabetes, CVD), cancer, hypertriglyceridemia, and hypercholesterolemia at baseline. They were also more likely to follow a special diet, and to take a nap after meals (siesta).

### 3.2. BRI and Incident Death

**Figure 2:**
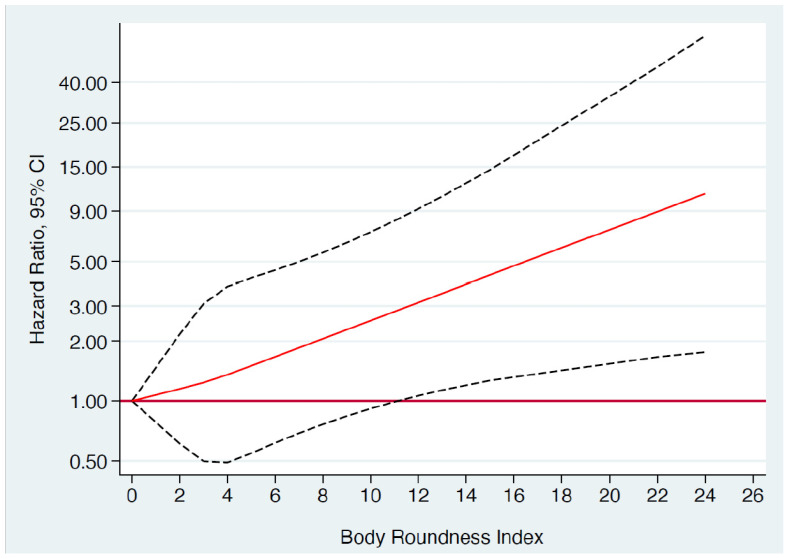
Restricted cubic splines dose–response associations: Adjusted HR and 95% CI for the risk of death according to quartiles of BRI among men and women in the SUN cohort. Adjusted (using inverse probability weighting) for parameters in Model 2: age, sex, marital status, smoking, pack/year, university education years, between meals snacking, adoption of special diet, prevalent hypertriglyceridemia, prevalent hypercholesterolemia, prevalent diseases (cancer, diabetes, cardiovascular disease), siesta, adherence to Mediterranean diet, television watching (h/d), total energy intake, Leisure-time physical activity (METs-h/wk), health-conscious score. Abbreviations: BRI: body roundness index; CI: confidence interval; HR: hazard ratio; SUN: Seguimiento Universidad de Navarra.

The results from the Cox model analyses of incident death according to BRI are presented in Table 2, with cumulative incidence curves shown in Figure 2. A strong and statistically significant direct relationship was observed between the baseline BRI quartiles and the incidence of death over the follow-up period across all models, demonstrating a monotonic trend. The continuous risk of incident death associated with each 2-unit increase in BRI was both strong and significant in the fully adjusted multivariate analyses, elevating the risk of death by 21% (HR 1.21; 95% CI 1.03, 1.43).

When we repeated these analyses considering participants over or under 60 years of age, we found that the significance of the association between baseline BRI and incident death remained significant only for those over 60 years of age (Table 3).

**Figure 3:**
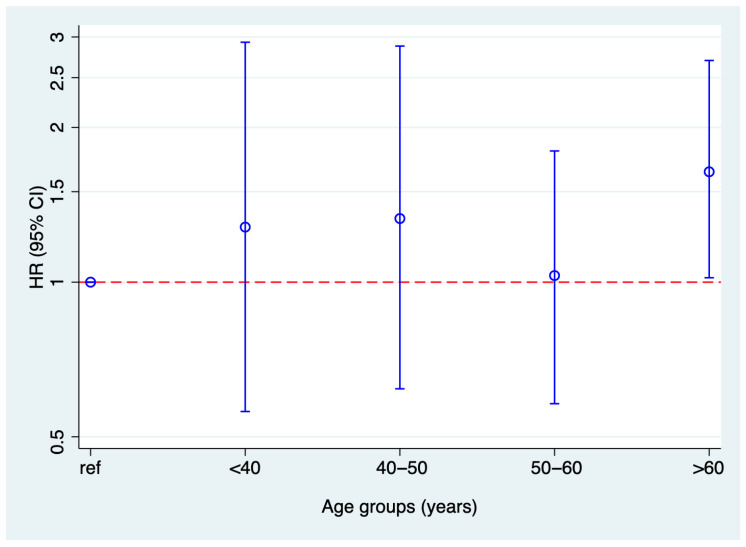
Risk of death in HRs and 95% CI in relation to BRI (between extreme quartiles) in different age groups. Abbreviations: BRI: body round index; CI: confidence interval; HR: hazard ratio.

Figure 3 displays the HR and 95% CI of incident death in relation to BRI in different age groups. There was no statistically significant interaction of BRI with age (*p* = 0.387).

Likewise, in the analysis examining the association between BRI and incident death, with a one SD (z-BRI) increase as a continuous variable, we observed a significantly higher risk of incident death. This was true when including the whole population, and again, only for participants older than 60 years across all models (Table 4).

Supplementary Table S2 shows the association between the quintiles of WtHR and incident mortality in men and women from the SUN cohort. As seen in the table, the association is significant only for model 1 (adjusted for age and sex) but loses significance when the other adjustment covariates are considered. Conversely, as shown in Supplementary Table S3, taking into account the z-WtHR, the association is significant even in the fully adjusted model.

Because WC is the simplest method to apply and is considered a vital sign in clinical practice [21], we also analyzed the association of WC with incident mortality. Supplementary Tables S4 and S5 show the results of these analyses, which were similar to those with WtHR: there is a lack of significance in the fully adjusted model for WC quintiles and statistical significance in the two models for z-WC.

**Figure 4:**
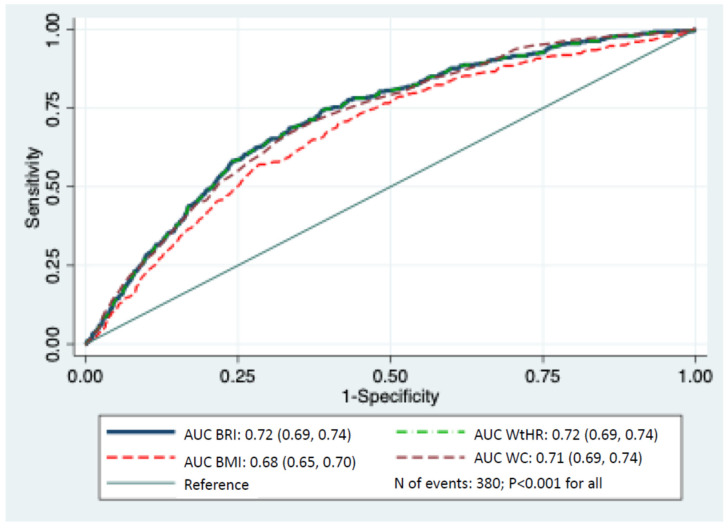
ROC analysis curves of BRI, WtHR, WC, and BMI for the prediction of death in men and women in the SUN (“Seguimiento Universidad de Navarra”) cohort, 2006–2024. Abbreviations: AUC: area under the curve; BMI: body mass index; BRI: body round index; CI: confidence interval; HR: hazard ratio; ROC: Receiver Operating Characteristic; WC: waist circumference; WtHR: waist-to-height ratio.

As regards the ROC curve analyses for BRI, WtHR, WC, and BMI in predicting death in both men and women, when comparing the AUC values of the curves, the prediction was similar and more accurate with BRI, WtHR, and WC than with BMI (*p* < 0.001) (Figure 4).

**Figure 5:**
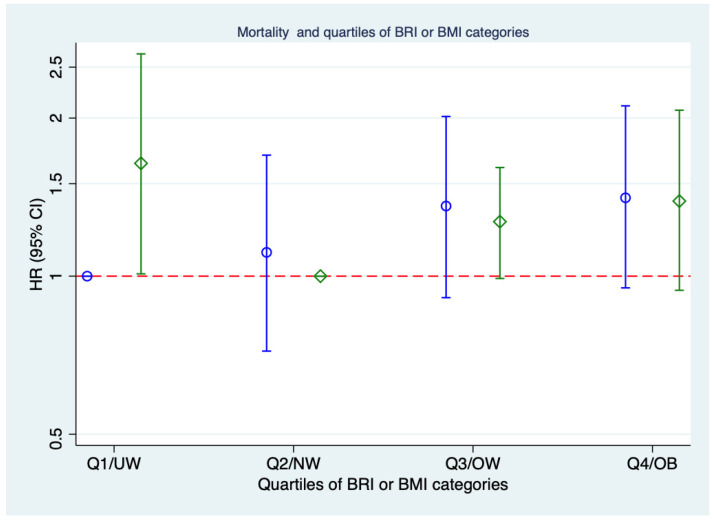
Risk of death according to quartiles of BRI (blue) and categories of BMI (green). Abbreviations: BMI: body mass index; BRI: body round index; CI: confidence interval; HR: hazard ratio; UW: underweight; NW: normoweight; OW: overweight; OB: obese; Q: quartile.

Multicollinearity among covariates was assessed using variance inflation factors, and no evidence of problematic collinearity was found. Table 5 and Figure 5 show the relationship of quartiles of BRI (A) and usual categories of BMI (B) in relation to the risk of mortality. As seen in the table, comparing the BRI quartiles and the BMI categories, the mortality risk in the upper BRI quartiles (Q3 and Q4) was slightly higher than in the upper BMI category.

### 3.3. Sensitivity Analyses

Several sensitivity analyses were conducted to assess the association between extreme quartiles of BRI and incident death in the fully adjusted multivariable models for the whole population as well as for participants older and younger than sixty years (Table 6). The results remained significant for people older than sixty years when we changed the allowable energy limits out of usual, as well as when we excluded participants with prevalent diabetes or hypertriglyceridemia at baseline. In the rest of the sensitivity analyses, although the trend was in the expected direction, they did not reach statistical significance, probably due to the wider width of the standard deviations.

## 4. Discussion

The current analysis of data from a large, well-characterized cohort of university graduates suggests a strong, positive and linear association of BRI with all-cause mortality in healthy adults aged over 60 years during a long follow-up period; the associations of z-WtHR and z-WC with incident mortality were also significant. These associations remained significant even after adjusting for multiple potential confounders and had a better independent association with death risk than BMI. Noteworthy, one of this factors is the adherence to the Mediterranean dietary pattern, which has been related to a lower risk of death in various studies [38,40,41,42]. Even including this strong confounder, the results were significant, suggesting that for participants with excess central adiposity, a better adherence to this high-quality dietary pattern is not able to reduce the mortality risk. This study is the first study to examine the longitudinal relationship between BRI and all-cause mortality in a Mediterranean population.

BMI is the most commonly used metric to classify individuals by weight status, but its accuracy in predicting body fat is limited [4,5,43]. Its use is controversial as it doesn’t reflect body composition or fat distribution. While BMI generally increases with adiposity, this relationship is weakened by variations in muscle mass and bone structure, meaning a high BMI may not always indicate excess fat or poor health. A recent retrospective analysis of 1999–2018 National Health Interview Survey data examined the link between self-reported BMI and mortality in a nationally representative U.S. adult sample (n = 554,332). Over a median 9-year follow-up, all-cause mortality risk was similar across most BMI categories—even among healthy never-smokers and after excluding early deaths. However, persons with BMI ≥ 30 kg/m^2^ showed a 21–108% higher mortality risk. Authors recommend including body composition and morbidity data in future research to better assess BMI-mortality relationships [5]. Furthermore, another study concluded that although available estimates account for several confounders in the BMI-mortality relationship (such as smoking, physical activity, and the racial/ethnic and gender composition of BMI groups), they do not control for other potential biases, including reverse causation and confounding due to variations in body shape associated with BMI [4].

While BMI has been useful in epidemiological studies, it may not accurately reflect individual health. According to the Lancet Diabetes & Endocrinology Commission [44], confirming excess adiposity with an additional measure (e.g., waist circumference) or direct fat assessment can help avoid misdiagnosis. A major limitation of BMI is its inability to assess body fat content or distribution. Waist circumference, in contrast, is now considered a valuable clinical vital sign [21].

Evidence shows that abdominal (central) obesity is more strongly associated with cardiometabolic and other chronic disease risks [21,45,46,47,48,49,50,51,52] than overall obesity. As such, central obesity measures may better reflect adiposity and be more closely linked to mortality risk than BMI. A meta-analysis by Jayedi et al. [53] involving 72 cohort studies (2.5M+ participants), found that central fatness indices were significantly and independently associated with higher all-cause mortality, suggesting their usefulness in assessing premature death risk.

The BRI proposed by Thomas et al. [22] estimates visceral fat relative to total body fat using waist circumference and height. It has proven more effective than other anthropometric measures in predicting risks for cardiometabolic disease [26,46,54,55,56,57], kidney disease [58,59], stroke [60], and cancer [61,62]. Additionally, only two longitudinal studies among Chinese general populations have demonstrated that a high BRI is significantly linked to an increased risk of both all-cause mortality and cardiovascular disease-specific mortality [56,63], and one specifically in patients with metabolic dysfunction-associated fatty liver disease (MAFLD) [64]. However, there is a lack of data on the relationship between BRI and mortality in populations from Mediterranean countries. Our study aimed to address this gap using data from a well-characterized and extensive Mediterranean cohort, which is the SUN project, observing a strong association of BRI, WtHR, and WC with all-cause mortality in this population.

The Mediterranean diet, known for its health benefits [65], originates from long-standing regional traditions but is increasingly threatened by globalized, Westernized eating habits [66]. In Mediterranean countries, adherence is declining in favour of diets high in sugary drinks, fast food, and ultra-processed foods, with reduced intake of vegetables, legumes, and whole grains [67]. This shift—driven by urbanization, food environment changes, and widespread availability of unexpensive, marketed products—is contributing to rising obesity and metabolic disorders [65], reinforcing the relevance of our findings linking indices of central adiposity, with mortality in this population.

We found that BRI was significantly associated with mortality in the overall population, but this association remained significant only in participants over 60. This focus is justified, as deaths linked to adiposity-related chronic diseases are less common under 60. However, the global obesity pandemic also heavily affects older adults [68], driven by high intake of energy-dense foods and sedentary lifestyles, even in developing countries with aging populations [69]. In older adults, obesity contributes to reduced physical function, poorer quality of life, and increased institutionalization rates [68,70].

The impact of overweight/obesity in older adults remains debated due to the “obesity paradox”—the idea that higher body fat may be protective in this age group, likely influenced by reverse causality [71]. Some studies have found that overweight individuals, especially older adults, had the lowest mortality rates across BMI categories [7,72]. This paradox is often seen in chronic inflammatory conditions like end-stage renal disease and heart failure, which are commonly associated with age-related muscle loss (sarcopenia) [73]. While obesity raises the risk of chronic diseases, some studies suggest obese patients may have higher survival once these conditions develop—possibly due to greater energy reserves and better nutrition [74,75]. However, obesity also promotes fat infiltration into muscle, reducing muscle quality and function and causing lipotoxicity, which impairs strength and leads to sarcopenia—a key risk factor for chronic disease and mortality in older adults [76,77,78,79]. Our findings highlight the role of central obesity, measured via BRI, WtHR, and WC which is linked to worse cardiometabolic and chronic disease outcomes [21,45,46,47,48,49,50,51,52], explaining the increased mortality risk, especially in older adults. This is notable given our cohort’s wide age range and the lack of prior longitudinal studies reporting such results in a Mediterranean population.

Supporting our findings on central obesity and mortality, the concept of “normal weight obesity” refers to individuals with normal BMI but high waist circumference or waist-to-hip ratio. A recent study of 7057 adults (≥65 years) with coronary artery disease found the highest mortality risk in those with normal BMI and central obesity—HR 1.29 (95% CI: 1.13–1.46) for high waist circumference, and HR 1.29 (95% CI: 1.12–1.50) for high waist-hip ratio [80]. These individuals often face elevated cardiometabolic risk but are frequently overlooked in clinical settings [81].

The strengths of this study include a large sample size, extensive follow-up, a prospective design, the ability to adjust for multiple potential confounders, and the high retention rate. Potential limitations are as follows: (i) The use of self-reported data, although variables such as self-reported weight, BMI, and waist circumference have been previously validated in sub-samples of our cohort [33,36]. (ii) The cohort is composed of highly educated participants with a lower prevalence of overweight/obesity and higher physical activity levels, which may explain the broader confidence intervals. However, we observed stronger associations between BRI and incident death compared to BMI. (iii) Some relevant confounding factors, such as socioeconomic status, education, disease, and access to medical care, were less prevalent in our study. Indeed, SUN project participants are university graduates from the University of Navarra and other Spanish institutions [30,31,32]. This selection reflects the epidemiological method of restriction, used to minimize confounding by socioeconomic status. As Rothman et al. note, restriction is an effective strategy to prevent or reduce confounding by known factors [82]. (iv) Another potential limitation is the possibility of residual confounding that must be acknowledge due to the lack of dietary quality assessment beyond Mediterranean diet score. Despite these cohort characteristics, the application of our findings to other populations should be based on biological mechanisms, rather than solely on statistical representativeness. Therefore, our results should be replicated in populations with different characteristics.

## 5. Conclusions

In our Mediterranean prospective multipurpose cohort, increased BRI—a validated tool for measuring central adiposity—was found to be positively, strongly, and independently associated with a higher risk of death, suggesting better discrimination than BMI. Moreover, simpler measures such as WtHR and WC were equally predictive of mortality, and all were superior to BMI for that outcome. These findings underscore the potential limitations of BMI and suggest that additional feasible assessment of central adiposity should be implemented in epidemiological studies and in clinical practice.

## Figures and Tables

**Table 1:** Baseline characteristics of men and women according to quartiles (Q) of Body Roundness Index (BRI) and according to categories of Body Mass Index (BMI) among participants in the SUN (“Seguimiento Universidad de Navarra”) cohort ^1^.

	BRI Quartiles			
	Q1	Q2	Q3	Q4
N	3164	3160	3165	3153
Limits of BRI (range) men	0.01, 3.5	3.5, 4.2	4.2, 5.0	5.0, 14.8
Limits of BRI (range) women	0.5, 2.2	2.2, 2.9	2.9, 3.8	3.8, 24.4
Mean BRI (men)	2.9 (0.5)	3.8 (0.2)	4.5 (0.2)	6.0 (1.0)
Mean BRI (women)	1.8 (0.3)	2.5 (0.2)	3.3 (0.3)	4.9 (1.3)
Age, y	33.0 (9.4)	37.1 (10.2)	41.3 (11.7)	44.7 (12.6)
Sex % women	60.2	60.2	60.3	60.2
Married, %	38.3	51.0	61.0	62.2
University education, y	5.0 (1.5)	5.1 (1.6)	5.1 (1.5)	5.1 (1.6)
BMI, kg/m^2^	22.2 (2.4)	22.5 (2.5)	23.7 (2.7)	26.4 (3.7)
*Smoking*				
-Never, %	58.8	52.4	46.1	39.6
-Current, %	23.2	23.4	23.6	26.7
-Former smoker, %	18.0	24.2	30.3	33.7
Smoking (pack/year)	2.5 (6.3)	3.7 (7.8)	5.1 (9.5)	7.3 (12.2)
Leisure-time physical activity, METs-h/wk	26.8 (27.2)	22.8 (22.8)	20.3 (20.8)	17.6 (18.3)
Television watching, h/d	1.5 (1.2)	1.6 (1.2)	1.6 (1.2)	1.7 (1.2)
Hypertension at baseline, %	4.7	7.3	11.2	20.5
Cancer at baseline, %	1.6	2.2	2.8	4.1
Diabetes at baseline, %	0.6	1.1	1.6	3.3
CVD at baseline, %	0.6	1.2	1.4	2.3
Hypertriglyceridemia at baseline, %	2.5	4.8	6.6	13.3
Hypercholesterolemia at baseline, %	10.2	15.7	19.0	25.6
Total energy intake, kcal/d	2585 (770)	2538 (759)	2464 (758)	2431 (785)
Adherence to Med Diet (score 1–9) ^2^	4.2 (1.8)	4.2 (1.8)	4.3 (1.8)	4.4 (1.8)
Adoption of special diets, %	5.7	6.1	7.6	12.0
Between-meal snacking, %	32.0	31.5	30.2	36.2
Siesta, %	47.6	53.2	57.4	58.7
Health conscious (score 1–10)	3.6 (1.7)	3.9 (1.8)	4.2 (1.9)	4.4 (1.9)
	**BMI Categories**			
	**Underweight** **<20** ** kg/m^2^**	**Normoweight** **20–25** ** kg/m^2^**	**Overweight** **25–30 kg/m^2^**	**Obesity** **>30 kg/m^2^**
N	1845	7111	3149	537
Mean BMI (men)	19.2 (0.7)	23.2 (1.3)	26.9 (1.3)	32.2 (2.2)
Mean BMI (women)	18.9 (0.8)	22.1 (1.4)	26.7 (1.3)	32.9 (2.8)
Age, y	31.9 (8.6)	37.7 (11.3)	45.0 (12.1)	46.3 (11.9)
Sex % women	95.9	67.2	28.7	29.4
Married, %	35.0	50.0	68.3	67.4
University education, y	4.9 (1.4)	5.1 (1.5)	5.3 (1.6)	5.1 (1.5)
BRI (men)	2.6 (0.7)	3.6 (0.8)	4.7 (1.0)	6.4 (1.5)
BRI (women)	2.2 (0.7)	3.1 (1.1)	4.5 (1.3)	6.3 (2.0)
*Smoking*				
-Never, %	59.4	51.5	40.2	37.4
-Current, %	25.7	24.1	23.5	25.1
-Former smoker, %	14.9	24.4	36.3	37.5
Smoking (pack/year)	2.4 (5.4)	4.3 (8.4)	8.6 (12.8)	10.8 (14.2)
Leisure-time physical activity, METs-h/wk	19.6 (20.8)	22.9 (23.9)	21.9 (21.9)	15.7 (17.0)
Television watching, h/d	1.6 (1.3)	1.6 (1.2)	1.7 (1.1)	1.8 (1.2)
Hypertension at baseline, %	2.4	7.3	19.7	36.7
Cancer at baseline, %	1.8	2.7	2.7	5.0
Diabetes at baseline, %	0.4	1.2	2.6	5.8
CVD at baseline, %	0.5	1.0	2.5	3.9
Hypertriglyceridemia at baseline, %	1.1	3.8	13.7	25.7
Hypercholesterolemia at baseline, %	9.7	14.5	26.4	34.1
Total energy intake, kcal/d	2574 (777)	2516 (763)	2539 (764)	2499 (856)
Adherence to Med Diet ^2^ (score 1–9)	4.0 (1.8)	4.2 (1.8)	4.4 (1.8)	4.4 (1.8)
Adoption of special diets, %	4.8	7.2	9.7	15.5
Between-meal snacking, %	33.9	31.4	31.4	47.7
Siesta, %	48.2	52.9	59.7	60.3
Health conscious (score 1–10)	3.7 (1.7)	4.0 (1.9)	4.2 (1.9)	4.3 (1.9)

^1^ Values are mean (SD) unless otherwise stated. ^2^ Mediterranean Diet Score, 0 to 9 points, according to reference [38]. Abbreviations: BMI: body mass index; BRI: body roundness index; CVD: cardiovascular disease; h/d: hours per day; kcal: kilocalories; Med: Mediterranean; MET: metabolic equivalent task; Q: quartile; SUN: Seguimiento Universidad de Navarra; y: years.

**Table 2:** Association between Quartiles of Body Roundness Index (BRI) and mortality among men and women in the SUN (“Seguimiento Universidad de Navarra”) cohort ^1^.

	BRI Quartiles					
	Q1	Q2	Q3	Q4	*p-Trend*	HR for +2 Units of BRI Increase
n	3164	3160	3165	3153		
BRI median (p25, p75) women	1.9 (1.6, 2.1)	2.5 (2.4, 2.7)	3.3 (3.1, 3.5)	4.5 (4.1, 5.2)		
BRI median (p25, p75) men	3.0 (2.7, 3.3)	3.8 (3.6, 4.0)	4.5 (4.4, 4.7)	5.7 (5.3, 6.4)		
Deaths	34	63	119	164		
Person-years of follow-up	55,107	55,400	54,814	52,999		
Crude rate (×10^−3^)	0.6	1.1	2.2	3.1		
Crude HR	1 (ref.)	1.04 (0.68, 1.58)	1.10 (0.74, 1.62)	1.12 (0.76, 1.65)	0.536	1.18 (1.01, 1.37)
Multivariate-adjusted HR ^2^ Model 1	1 (ref.)	1.11 (0.73, 1.69)	1.38 (0.93, 2.06)	1.53 (1.04, 2.27)	0.011	1.29 (1.10, 1.51)
Multivariate-adjusted HR ^3^ Model 2	1 (ref.)	1.11 (0.72, 1.70)	1.36 (0.91, 2.04)	1.41 (0.95, 2.11)	0.065	**1.21 (1.03, 1.43)**

^1^ Values are HR estimated with Cox regression and 95% confidence intervals (CI). ^2^ Model 1: adjusted for age and sex. ^3^ Model 2: HR adjusted for factors in Model 1 plus marital status, smoking, pack/year, university education years, between meals snacking, adoption of special diet, prevalent hypertriglyceridemia, prevalent hypercholesterolemia, prevalent diseases (cancer, diabetes, cardiovascular disease), siesta, adherence to Mediterranean diet, television watching (h/d), total energy intake, Leisure-time physical activity (METs-h/wk), health-conscious score. Abbreviations: BRI: body roundness index; HR: hazard ratio; Q: quartile; SUN: Seguimiento Universidad de Navarra.

**Table 3:** Association between Quartiles of Body Roundness Index (BRI) and mortality among men and women stratified by age in the SUN (“Seguimiento Universidad de Navarra”) cohort ^1^.

	BRI Quartiles					
	Q1	Q2	Q3	Q4	*p-Trend*	HR for +2 Units of BRI Increase
Age < 60 y						
n	3035	2963	2984	2982		
BRI median (p25, p75) women	1.9 (1.6, 2.1)	2.5 (2.4, 2.7)	3.2 (3.0, 3.5)	4.5 (4.0, 5.2)		
BRI median (p25, p75) men	3.0 (2.6, 3.2)	3.7 (2.6, 3.9)	4.4 (4.2, 4.6)	5.5 (5.2, 6.3)		
Deaths	28	40	65	84		
Person-years of follow-up	52,904	52,007	52,122	50,640		
Crude rate (×10^−3^)	0.5	0.8	1.2	1.7		
Crude HR	1 (ref.)	0.87 (0.53, 1.41)	1.00 (0.64, 1.58)	1.01 (0.65, 1.58)	0.653	1.14 (0.94, 1.38)
Multivariate-adjusted HR ^2^ Model 1	1 (ref.)	1.01 (0.62, 1.65)	1.30 (0.82, 2.06)	1.41 (0.90, 2.22)	0.059	1.23 (1.00, 1.50)
Multivariate-adjusted HR ^3^ Model 2	1 (ref.)	1.01 (0.62, 1.66)	1.24 (0.78, 1.98)	1.22 (0.77, 1.95)	0.332	1.10 (0.88, 1.36)
Age ≥ 60 y						
n	171	169	169	169		
BRI median (p25, p75) women	2.9 (2.6, 3.1)	3.7 (3.6, 3.9)	4.5 (4.2, 4.6)	5.5 (5.2, 6.3)		
BRI median (p25, p75) men	3.8 (3.5, 4.1)	4.6 (4.5, 4.8)	5.3 (5.2, 5.5)	6.1 (5.9, 6.9)		
Deaths	32	50	37	44		
Person-years of follow-up	2779	2680	2673	2513		
Crude rate (×10^−3^)	11.5	18.7	13.8	17.5		
Crude HR	1 (ref.)	1.46 (0.93, 2.29)	1.23 (0.76, 1.99)	1.46 (0.91, 2.32)	0.202	**1.38 (1.07, 1.77)**
Multivariate-adjusted HR ^2^ Model 1	1 (ref.)	1.48 (0.94, 2.32)	1.23 (0.76, 1.99)	1.70 (1.06, 2.72)	0.060	**1.41 (1.08, 1.83)**
Multivariate-adjusted HR ^3^ Model 2	1 (ref.)	1.61 (1.00, 2.60)	1.19 (0.71, 2.00)	** 1.64 (1.00, 2.70) **	0.132	** 1.31 (1.00, 1.72) **

^1^ Values are HR estimated with Cox regression and 95% confidence intervals (CI). ^2^ Model 1: adjusted for age and sex. ^3^ Model 2: HR adjusted for factors in Model 1 plus marital status, smoking, pack/year, university education years, between meals snacking, adoption of special diet, prevalent hypertriglyceridemia, prevalent hypercholesterolemia, prevalent diseases (cancer, diabetes, cardiovascular disease), siesta, adherence to Mediterranean diet, television watching (h/d), total energy intake, Leisure-time physical activity (METs-h/wk), health-conscious score. Bold indicates the most significant results. Abbreviations: BRI: body roundness index; HR: hazard ratio; Q: quartile; SUN: Seguimiento Universidad de Navarra; y: years.

**Table 4:** Association between z-Body Roundness Index (BRI) (i.e., for each SD, as a continuous variable) and mortality among men and women in the SUN (“Seguimiento Universidad de Navarra”) cohort ^1^.

	All		Age < 60 y		Age ≥ 60 y	
	z-BRI	*p*	z-BRI	*p*	z-BRI	*p*
n	12,642		11,964		678	
Persons-year	218,319		207,674		10,645	
Crude rate (×10^−3^)	1.7		1.0		15,3	
Crude HR	1.06 (0.96, 1.18)	0.245	1.04 (0.91, 1.19)	0.604	1.15 (0.97, 1.36)	0.113
Multivariate-adjusted HR ^2^ Model 1	** 1.17 (1.06, 1.30) **	** 0.003 **	1.14 (1.00, 1.30)	0.052	** 1.24 (1.04, 1.48) **	** 0.016 **
Multivariate-adjusted HR ^3^ Model 2	** 1.13 (1.01, 1.26) **	** 0.027 **	1.06 (0.92, 1.22)	0.447	** 1.20 (1.00, 1.43) **	** 0.046 **

^1^ Values are HR estimated with Cox regression and 95% confidence intervals (CI). ^2^ Model 1: adjusted for age and sex. ^3^ Model 2: HR adjusted for factors in Model 1 plus marital status, smoking, pack/year, university education years, between meals snacking, adoption of special diet, prevalent hypertriglyceridemia, prevalent hypercholesterolemia, prevalent diseases (cancer, diabetes, cardiovascular disease), siesta, adherence to Mediterranean diet, television watching (h/d), total energy intake, Leisure-time physical activity (METs-h/wk), health-conscious score. Bold indicates the most significant results. Abbreviations: BRI: body roundness index; HR: hazard ratio; SUN: Seguimiento Universidad de Navarra; y: years.

**Table 5:** Multivariate adjusted HR of quartiles (Q) of Body Roundness Index (BRI) (**A**) and categories of BMI (**B**) and mortality risk among men and women in the SUN (“Seguimiento Universidad de Navarra”) cohort ^1^.

**A**				
**BRI Quartiles**	**Q1**	**Q2**	**Q3**	**Q4**
BMI mean (SD)	22.2 (2.4)	22.5 (2.5)	23.7 (2.7)	26.4 (3.7)
Mortality HR	1 (ref.)	1.11 (0.72, 1.70)	1.36 (0.91, 2.04)	1.41 (0.95, 2.11)
**B**				
**BMI Categories**	**Underweight** **(<20** ** kg/m^2^** **)**	**Normoweight** **(20–25** ** kg/m^2^** **)**	**Overweight** **(25–30** ** kg/m^2^** **)**	**Obese** **(>30** ** kg/m^2^** **)**
BRI mean (SD)	2.21 (0.74)	3.10 (1.06)	4.49 (1.27)	6.31 (1.98)
BRI limits	0.47–8.63	0.64–24.36	0.84–11.79	1.59–13.16
Mortality HR	1.64 (1.01, 2.65)	1 (ref.)	1.27 (0.99, 1.61)	1.39 (0.94, 2.07)

^1^ Values are HR estimated with Cox regression and 95% confidence intervals (CI). HR adjusted for age, sex, marital status, smoking, pack/year, university education years, between meals snacking, adoption of special diet, prevalent hypertriglyceridemia, prevalent hypercholesterolemia, prevalent diseases (cancer, diabetes, cardiovascular disease), siesta, adherence to Mediterranean diet, television watching (h/d), total energy intake, Leisure-time physical activity (METs-h/wk), health-conscious score. Abbreviations: BRI: body roundness index; HR: hazard ratio; Q: quartile; SUN: Seguimiento Universidad de Navarra.

**Table 6:** Sensitivity analyses: Multivariable-adjusted Hazard Ratios of mortality associated with Body Roundness Index (BRI) between extreme quartiles, among men and women in the SUN (“Seguimiento Universidad de Navarra”) cohort ^1^.

	All			Age ≥ 60 y		
	n	Death n	HR (95% CI) ^2^	n	Death n	HR (95% CI) ^2^
Main analysis ^2^	12,642	380	1.41 (0.95, 2.11)	678	163	1.64 (1.00, 2.70)
Changing allowable energy limits out of usual ^2^	11,708	360	1.32 (0.88, 2.00)	528	133	2.15 (1.18, 389)
Including only never smokers ^2^	6189	110	1.19 (0.60, 2.36)	222	47	2.12 (0.75, 5.97)
Excluding participants with prevalent hypertension ^2^	11,261	238	1.32 (0.83, 2.09)	-	-	-
Excluding participants with prevalent diabetes ^2^	12,434	352	1.51 (1.00, 2.28)	610	144	1.93 (1.14, 3.28)
Excluding participants with prevalent cancer ^2^	12,303	344	1.40 (0.93, 2.13)	619	142	1.64 (0.95, 2.82)
Excluding participants with prevalent hypertriglyceridemia ^2^	11,785	308	1.54 (0.99, 2.40)	532	127	1.82 (1.02, 3.25)
Excluding participants with prevalent hypercholesterolemia ^2^	10,416	257	1.49 (0.95, 2.35)	407	104	1.46 (0.76, 2.77)
Excluding underweight participants	10,797	358	1.61 (1.02, 2.55)	672	163	1.72 (0.70, 4.24)

^1^ Values are HR estimated with Cox regression and 95% confidence intervals (CI). ^2^ HR adjusted for age, sex, marital status, smoking, pack/year, university education years, between meals snacking, adoption of special diet, prevalent hypertriglyceridemia, prevalent hypercholesterolemia, prevalent diseases (cancer, diabetes, cardiovascular disease), siesta, adherence to Mediterranean diet, television watching (h/d), total energy intake, Leisure-time physical activity (METs-h/wk), health-conscious score. Abbreviations: BRI: body roundness index; HR: hazard ratio; Q: quartile; SUN: Seguimiento Universidad de Navarra; y: years.

## Data Availability

The data that support the findings of this study are available from the SUN Project at sun@unav.es upon reasonable request.

## Supplementary Materials

The following supporting information can be downloaded at: https://www.mdpi.com/article/10.3390/geriatrics10060135/s1.

## Abbreviations

The following abbreviations are used in this manuscript:

AUC	Area Under the Curve
BMI	Body Mass Index
BRI	Body Roundness Index
CI	Confidence Interval
HR	Hazard Ratio
MED	Mediterranean
MET	Metabolic Equivalent of Task
NHANES	National Health and Nutrition Examination Survey
Q	Quartile
ROC	Receiver Operating Characteristic
SD	Standard Deviation
SUN	Seguimiento Universidad de Navarra
WC	Waist circumference
WtHR	Waist-to-height ratio
