# Supplementary material for: Central Adiposity Assessed with Body Roundness Index and Mortality: The Seguimiento Universidad de Navarra Prospective Cohort
Source: Geriatrics (Basel). 2025 Oct 23;10(6):135. doi: 10.3390/geriatrics10060135 (PMC12641651; doi:10.3390/geriatrics10060135)
Supplement: Supplementary file 1 [file geriatrics-10-00135-s001.zip › geriatrics-3782922-supplementary.pdf]

Suppl. Table S1. Baseline characteristics of men and women included and excluded from the present analyses among participants in the SUN ("Seguimiento Universidad de Navarra") cohort[1].

	Included	Excluded
N	12642	9952
Mean BRI (men and women)	3.6 (1.4)	3.6 (1.6)
Limits of BRI (range) men	0.01, 14.8	0.04, 19.9*
Limits of BRI (range) women	0.5, 24.4	0.6, 17.7*
Mean BRI (men)	4.3 (1.3)	4.4 (1.6)
Mean BRI (women)	3.1 (1.3)	3.2 (1.4)
Age, y	39.0 (12.0)	36.3 (12.6)
Sex % women	60.2	62.9
Married, %	53.1	43.8
University education, y	5.1 (1.5)	5.0 (1.5)
BMI, kg/m^2	23.5 (3.4)	23.5 (3.7)
Smoking		
- Never, %	49.2	49.0
- Current, %	24.2	27.9
- Former smoker, %	26.5	23.1
Smoking (pack/year)	4.6 (9.4)	4.2 (9.2)

Leisure-time physical activity, METs-h/wk	21.9 (22.8)	22.2 (24.0)
Television watching, h/d	1.6 (1.2)	1.6 (1.2)
Hypertension at baseline, %	10.9	10.9
Cancer at baseline, %	2.7	2.3
Diabetes at baseline, %	1.7	2.1
CVD at baseline, %	1.4	1.7
Hypertriglyceridemia at baseline, %	6.8	6.7
Hypercholesterolemia at baseline, %	17.6	15.8
Total energy intake, kcal/d	2504 (770)	2479 (789)
Adherence to Med Diet[2] (1-9)	4.3 (1.8)	4.2 (1.8)
Adoption of special diets, %	7.8	8.5
Between-meal snacking, %	32.5	37.8
Siesta, %	54.2	56.3
Health conscious (1-10)	4.0 (1.9)	3.9 (1.9)

*When data were available
CVD: cardiovascular disease; Med: Mediterranean; MET: metabolic equivalent task.
[1] Values are mean (SD) unless otherwise stated.
[2] Mediterranean Diet Score, 0 to 9 points, according to reference [39].

Suppl. Table S2. Association between Quintiles of waist to height ratio (WtHR) and mortality among men and women in the SUN ("Seguimiento Universidad de Navarra") cohort[1].

	WtHR quintiles					
	Q1	Q2	Q3	Q4	Q5	*p* trend
n	2536	2523	2581	2485	2517	
WtHR median (p25, p75) women	0.41 (0.39, 0.42)	0.44 (0.44, 0.45)	0.47 (0.47, 0.48)	0.51 (0.50, 0.52)	0.57 (0.55, 0.60)	
WtHR median (p25, p75) men	0.47 (0.46, 0.49)	0.51 (0.51, 0.52)	0.54 (0.53, 0.55)	0.57 (0.56, 0.58)	0.62 (0.60, 0.65)	
Deaths	30	40	69	104	137	
Person-years of follow-up	44,108	44,127	45,168	42,732	42,184	
Crude rate ($x10^{-3}$)	0.6	0.9	1.5	2.4	3.2	
Crude HR	1 (ref.)	0.87 (0.53, 1.40)	0.91 (0.59, 1.41)	0.98 (0.65, 1.49)	1.00 (0.66, 1.50)	0.579
Multivariate-adjusted HR[2] Model 1	1 (ref.)	0.90 (0.56, 1.45)	1.06 (0.69, 1.66)	1.24 (0.81, 1.90)	1.35 (0.89, 2.06)	**0.018**
Multivariate-adjusted HR[3] Model 2	1 (ref.)	0.93 (0.57, 1.51)	1.08 (0.69, 1.68)	1.23 (0.80, 1.89)	1.25 (0.82, 1.92)	0.108

[1] Values are HR estimated with Cox regression and 95% confidence intervals (CI).

[2] Model 1: adjusted for age and sex.

[3] Model 2: HR adjusted for factors in Model 1 plus marital status, smoking, pack/year, university education years, between meals snacking, adoption of special diet, prevalent hypertriglyceridemia, prevalent hypercholesterolemia, prevalent diseases (cancer, diabetes, cardiovascular disease), siesta, adherence to Mediterranean diet, television watching (h/d), total energy intake, Leisure-time physical activity (METs-h/wk), health-conscious score.

Abbreviations: WtHR: Weight to height ratio; HR: hazard ratio; Q: quartile; SUN: Seguimiento Universidad de Navarra.

Suppl Table S3. Association between z-waist to height ratio (WtHR) (i.e., for each SD, as a continuous variable) and mortality among men and women in the SUN ("Seguimiento Universidad de Navarra") cohort[1].

	All	
	z-WtHR	p
n	12642	
Persons-year	218,319	
Crude rate ($x10^{-3}$)	1.7	
Crude HR	1.06 (0.95, 1.19)	0.245
Multivariate-adjusted HR[2] Model 1	**1.18 (1.05, 1.32)**	**0.003**
Multivariate-adjusted HR[3] Model 2	**1.14 (1.01, 1.27)**	**0.027**

[1] Values are HR estimated with Cox regression and 95% confidence intervals (CI).

[2] Model 1: adjusted for age and sex.

[3] Model 2: HR adjusted for factors in Model 1 plus marital status, smoking, pack/year, university education years, between meals snacking, adoption of special diet, prevalent hypertriglyceridemia, prevalent hypercholesterolemia, prevalent diseases (cancer, diabetes, cardiovascular disease), siesta, adherence to Mediterranean diet, television watching (h/d), total energy intake, Leisure-time physical activity (METs-h/wk), health-conscious score. Bold indicates the most significant results.

Abbreviations: BRI: body roundness index; HR: hazard ratio; SUN: Seguimiento Universidad de Navarra; y: years.

Suppl. Table S4. Association between Quintiles of waist circumference (WC) and mortality among men and women in the SUN ("Seguimiento Universidad de Navarra") cohort[1].

	Waist circumference quintiles					
	Q1	Q2	Q3	Q4	Q5	*p* trend
n	2809	2676	2379	2439	2339	
WC, cm median (p25, p75) women	68 (65, 69)	73 (72, 75)	78 (77, 80)	84 (82, 85)	93 (90, 99)	
WC, cm median (p25, p75) men	84 (80, 86)	91 (89, 92)	95 (94, 96)	100 (99, 102)	109 (105, 114)	
Deaths	33	59	77	96	115	
Person-years of follow-up	48,692	46,889	41,691	41,836	39,210	
Crude rate (x10^{-3})	0.7	1.2	1.8	2.3	2.9	
Crude HR	1 (ref.)	1.05 (0.68, 1.62)	1.17 (0.77, 1.76)	1.06 (0.72, 1.60)	1.22 (0.82, 1.82)	0.263
Multivariate-adjusted HR[2] Model 1	1 (ref.)	1.09 (0.71, 1.67)	1.34 (0.88, 2.04)	1.32 (0.88, 1.99)	1.57 (1.05, 234)	**0.009**
Multivariate-adjusted HR[3] Model 2	1 (ref.)	1.05 (0.68, 1.63)	1.31 (0.86, 2.00)	1.25 (0.82, 1.90)	1.40 (0.93, 2.12)	0.071

[1] Values are HR estimated with Cox regression and 95% confidence intervals (CI).

[2] Model 1: adjusted for age and sex.

[3] Model 2: HR adjusted for factors in Model 1 plus marital status, smoking, pack/year, university education years, between meals snacking, adoption of special diet, prevalent hypertriglyceridemia, prevalent hypercholesterolemia, prevalent diseases (cancer, diabetes, cardiovascular disease), siesta, adherence to Mediterranean diet, television watching (h/d), total energy intake, Leisure-time physical activity (METs-h/wk), health-conscious score.

Abbreviations: WC waist circumference; HR: hazard ratio; Q: quartile; SUN: Seguimiento Universidad de Navarra.

Suppl Table S5. Association between z-waist circumference (WC) (i.e., for each SD, as a continuous variable) and mortality among men and women in the SUN ("Seguimiento Universidad de Navarra") cohort[1].

	All	
	z-WC	p
n	12642	
Persons-year	218,319	
Crude rate ($x10^{-3}$)	1.7	
Crude HR	1.11 (0.99, 1.24)	0.068
Multivariate-adjusted HR[2] Model 1	**1.20 (1.07, 1.33)**	**0.001**
Multivariate-adjusted HR[3] Model 2	**1.16 (1.04, 1.30)**	**0.010**

[1] Values are HR estimated with Cox regression and 95% confidence intervals (CI).

[2] Model 1: adjusted for age and sex.

[3] Model 2: HR adjusted for factors in Model 1 plus marital status, smoking, pack/year, university education years, between meals snacking, adoption of special diet, prevalent hypertriglyceridemia, prevalent hypercholesterolemia, prevalent diseases (cancer, diabetes, cardiovascular disease), siesta, adherence to Mediterranean diet, television watching (h/d), total energy intake, Leisure-time physical activity (METs-h/wk), health-conscious score. Bold indicates the most significant results.

Abbreviations: BRI: body roundness index; HR: hazard ratio; SUN: Seguimiento Universidad de Navarra; y: years.
